# Identification of osimertinib-resistant *EGFR* L792 mutations by cfDNA sequencing: oncogenic activity assessment and prevalence in large cfDNA cohort

**DOI:** 10.1186/s40164-019-0148-7

**Published:** 2019-10-11

**Authors:** Stephen R. Fairclough, Lesli A. Kiedrowski, Jessica J. Lin, Ori Zelichov, Gabi Tarcic, Thomas E. Stinchcombe, Justin I. Odegaard, Richard B. Lanman, Alice T. Shaw, Rebecca J. Nagy

**Affiliations:** 1 Guardant Health, Inc., 505 Penobscot Dr, Redwood City, CA 94063 USA; 2 Massachusetts General Hospital Cancer Center and Department of Medicine, Massachusetts General Hospital, 55 Fruit St, Boston, MA 02114 USA; 3 NovellusDx, Jerusalem Biopark, Hadassah Ein-Kerem Medical Center Campus, Jerusalem, Israel; 4 Duke Cancer Institute, DUMC 3198, 25178 Morris Building, Durham, NC 27710 USA

**Keywords:** Cell-free DNA, cfDNA, Genomic evolution, Acquired resistance, Osimertinib, Guardant360, Functional studies

## Abstract

Cell-free DNA (cfDNA) next-generation sequencing has the potential to capture tumor heterogeneity and genomic evolution under treatment pressure in a non-invasive manner. Here, we report the detection of *EGFR* L792 mutations, a non-covalent mechanism of osimertinib resistance, using Guardant360 cfDNA testing in a patient with metastatic *EGFR*-mutant non-small cell lung cancer (NSCLC) whose disease progressed on osimertinib. We subsequently analyzed a large cohort of over 1800 additional patient samples harboring an *EGFR* T790M mutation and identified a concomitant L792 mutation in a total of 22 (1.2%) cases. In vitro functional assays demonstrated that the *EGFR* L858R/T790M/L792F/H mutations conferred intermediate-level resistance to osimertinib. Further understanding of potential acquired resistance mechanisms to targeted therapy may help inform treatment strategy in *EGFR*-mutant NSCLC.

## Background

Epidermal growth factor receptor (*EGFR*)-mutant non-small cell lung cancer (NSCLC) is a distinct molecular subtype with sensitivity to EGFR-selective tyrosine kinase inhibitors (TKIs) [1–4]. However, tumors invariably develop resistance to these EGFR TKIs, mediated by on-target genetic alterations within the *EGFR* tyrosine kinase domain, *EGFR*-independent mechanisms, or small cell transformation [5, 6]. In initial reports of acquired resistance to first-generation EGFR TKIs erlotinib and gefitinib, 50–60% of cases harbored an *EGFR* T790M gatekeeper mutation [5, 6]. Osimertinib, an irreversible, third-generation EGFR inhibitor, was developed to target T790M mutation-positive, first-generation TKI-refractory tumors and demonstrated robust efficacy with objective response rates of 61–71% among T790M-positive NSCLC patients [7–9]. More recently, osimertinib became the new standard initial therapy in advanced *EGFR*-mutant NSCLC [10]. Despite its efficacy, patients acquire resistance to osimertinib through various mechanisms including *EGFR* C797S mutations which eliminate the covalent bonding site for osimertinib, and amplification of *MET* or *ERBB2* (HER2), among others [11–13]. The prevalence of C797S mutations may differ depending on the clinical setting and is more common in patients with a pre-existing T790M mutation [14, 15]. Serial assessment of the molecular characteristics of *EGFR*-mutant NSCLC with each line of therapy will assist in understanding the evolution of on- and off-target mechanisms of resistance and can help guide the development of new therapeutic strategies for patients with resistant disease.

Historically, tumor tissue biopsies have been standard for detection of resistance mechanisms. However, tissue biopsies are inevitably limited by their invasive procedural risk, high cost, treatment delays related to procedure and processing, and inability to capture spatial tumor heterogeneity. In contrast, plasma cell-free DNA (cfDNA) next-generation sequencing (NGS) from peripheral blood allows for safe, global, and repeated longitudinal assessment of mutation dynamics throughout the course of disease and treatment. Therefore, this approach has the potential to accelerate our understanding of TKI resistance.

Here, we report the detection of *EGFR* L792 resistance mutations via cfDNA sequencing in a patient progressing on osimertinib, their prevalence in a large clinically tested NSCLC cfDNA cohort, and in vitro functional characterization.

## Case report

**Fig. 1:**
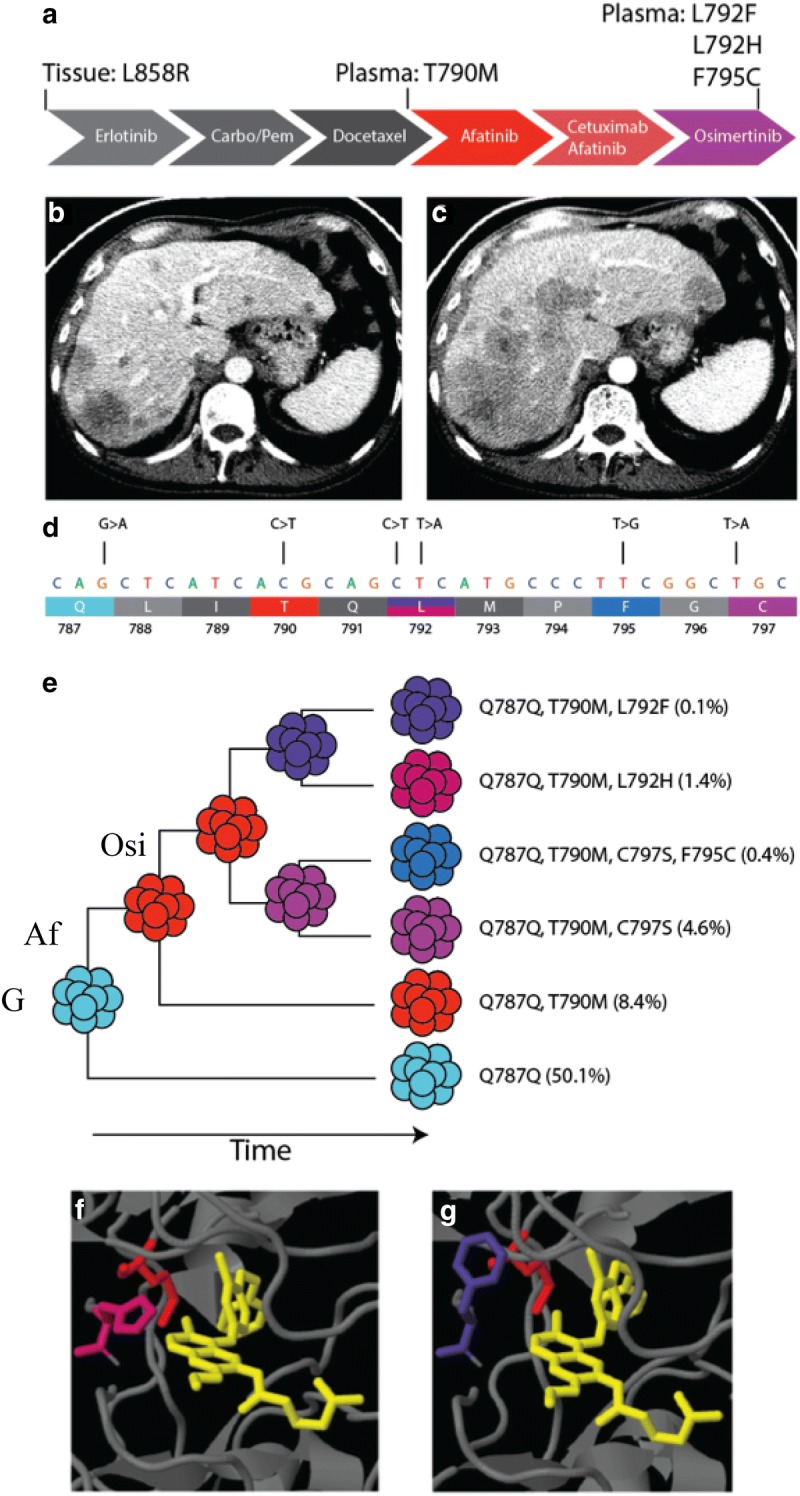
Identification of *EGFR* L792F and L792H mutations by cfDNA sequencing in osimertinib-resistant NSCLC. Somatic cfDNA profiling of a patient progressing on osimertinib revealed the known resistance mutation C797S, as well as novel *EGFR* mutations L792F, L792H, and F795C. **a** Patient treatment history. **b** Abdominal CT 2 months after initiation of osimertinib, showing stable hepatic metastases. **c** Abdominal CT 7 months after initiation on osimertinib, showing multifocal progression throughout the liver. **d** Schema of somatic *EGFR* mutations identified by cfDNA NGS and corresponding predicted amino acid alterations. **e** Presumptive evolutionary history inferred by dollo parsimony analysis of phased mutations. *G* germline, *Af* afatinib, *Osi* osimertinib. **f** The structural location of L792H mutation (magenta) in EGFR relative to T790M (red) and bound TKI (yellow). **g** The structural location of L792F mutation (purple) in EGFR relative to T790M (red) and bound TKI (yellow)

A 68-year-old male former smoker with *EGFR* L858R-mutant metastatic NSCLC presented after progression on multiple lines of therapy, including first-line erlotinib, carboplatin/pemetrexed, docetaxel, followed by afatinib. cfDNA droplet digital PCR identified the *EGFR* T790M resistance mutation (Fig. 1a). After a short course of cetuximab + afatinib, the patient began osimertinib with disease control; 7 months later, imaging demonstrated progressive disease (Fig. 1b, c). At this time, cfDNA profiling was performed using Guardant360, a highly sensitive and ultra-specific 70-gene NGS panel, which interrogated the entire *EGFR* coding sequence for SNVs, indels, and gene amplification (Additional file 1: Figure S1) [16]. Twelve somatic alterations were identified, including seven alterations in *EGFR* (Additional file 1: Table S1). The original L858R activating *EGFR* mutation was present at a variant allele fraction (VAF) of 16.9%, and the T790M mutation was present at a VAF of 8.4%. In addition, this analysis revealed *EGFR* C797S (4.6%) and L718Q (0.7%) mutations, both of which have been previously reported as osimertinib resistance mechanisms [11, 12, 17, 18]. Interestingly, three additional tyrosine kinase domain mutations were identified: L792H (1.4%), F795C (0.4%), and L792F (0.1%) (Fig. 1d). While *EGFR* L792 mutations have recently been reported as resistance mechanisms to osimertinib [19, 20], at the time of this patient’s clinical presentation these were novel findings which spurred further investigation.

Given their genomic proximity, the T790M and C797S mutations were phased to determine allelic origin and found to be in *cis*, and the F795C mutation appeared on that allele. In contrast, the L792H and L792F variants were in *cis* to T790M but arose in *trans* to C797S and to each other. While multiple tissue biopsies over time were not available to determine the temporal sequence of mutational emergence, when mapped against the patient’s treatment history the clonal phylogeny of these *EGFR* alleles suggested that at least the L792H and L792F mutations arose during osimertinib treatment at the same branch point as the known osimertinib resistance mutation C797S (Fig. 1e). Moreover, structural modeling indicated that each mutation affects a residue that impinges on the ATP-binding pocket (Fig. 1f, g).

## Prevalence in a large cfDNA cohort

Given the evidence linking L792H and L792F mutations to osimertinib resistance, the Guardant360 clinical genomic cfDNA database of *EGFR*-mutant lung cancer samples from 10/14/2015 through 2/4/2019 was retrospectively analyzed to investigate the prevalence of these alterations. 1851 patients were identified whose samples contained *EGFR* T790M mutations. While detailed clinical information including treatment history is unavailable for this cohort, somatic *EGFR* T790M mutations are rare outside of the setting of resistance to early-generation TKIs [21]. Of these patients, 22 (1.2%) also had at least one nonsynonymous *EGFR* L792 alteration identified (Table 1, Additional file 1: Table S2). Of these L792-positive patients, 11 (50%) also had at least one *EGFR* C797S clone identified. Notably, of the overall cohort of *EGFR* T790M-positive lung cancer patients, 151 (8.2%) had *EGFR* C797S identified in their clinical cfDNA testing, considerably more frequent versus the 1.2% prevalence of L792 variants.

**Table 1:** Nonsynonymous *EGFR* L792 alterations co-occurring with *EGFR* T790M mutations identified in the Guardant360 database of patients with lung cancer

Alteration(s)	Number of patients
L792H	9
L792F	6
L792P	2
L792R	1
L792H and L792V	2
L792V and L792F	1
L792H and L792F	1^a^

^a^Initial patient whose case is described in detail

Besides the initial case described above, only one other patient was found to have a nonsynonymous *EGFR* F795 alteration in conjunction with an L792 mutation; this patient’s sample had 14 nonsynonymous *EGFR* alterations (Additional file 1: Table S2). One additional patient’s sample harbored an *EGFR* L792R alteration in the absence of a co-occurring *EGFR* T790M mutation; six other nonsynonymous *EGFR* alterations were detected in this sample (L717V, L718Q, G796S, C797S, G796R, and S1036R).

Phasing analysis was performed on 27 samples from the 22 unique patients containing an L792F/H/V/P/R or F795C/L mutation. As in the initial case described above, the L792 F/H/V/P/R and F795C/L alterations were invariably present subclonal to and frequently in *cis* with *EGFR* T790M, but independent of one another, C797S, and other osimertinib resistance alterations (Additional file 1: Table S3). The recurrence of these mutations across multiple patients supports the hypothesis that these variants confer a selective advantage compatible with osimertinib resistance. However, the relatively low frequency with which these variants are observed and lower VAFs at which they occur suggest that this advantage may be less potent than that conferred by C797S.

## Functional studies

**Fig. 2:**
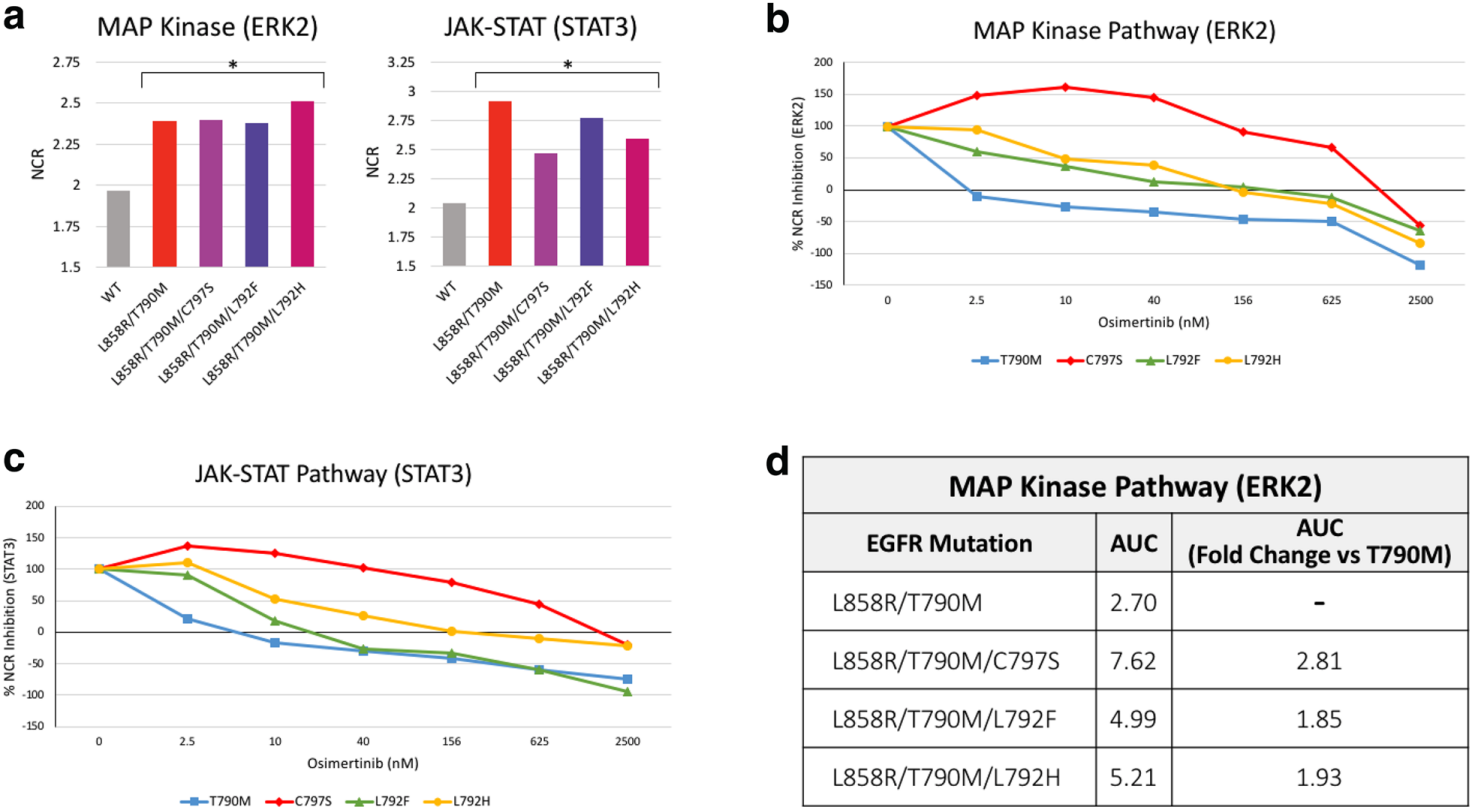
Functional assessment of L792F/H *EGFR* mutations and sensitivity to osimertinib. Functional evaluation of the L792F/H mutations was performed using an in vitro assay which uses high-content microscopy to assess activation of oncogenic signaling pathways represented by the nuclear-to cytoplasmic ratio (NCR) of signaling pathway reporters. Activity was assessed for the MAP Kinase pathway (ERK2-reporter) and JAK-STAT pathway (STAT3-reporter). **a** Baseline functional activity of *EGFR* mutations compared to wild-type *EGFR*. Values are average NCR for each condition, *p < 0.05 (students *T* test) with bracket indicating that the difference in activation between WT *EGFR* and each of the four mutations is significant. Presented is a representative experiment of 3 repeats. **b**, **c** Sensitivity to osimertinib was measured in escalating nMol concentrations. Values are the mean percentage (%) activation of ΔNCR (*MTtx* *−* *WTut*)/(*MTut* *−* *WTut*) normalized for each condition. 100% is the over-activation due to MT construct activity and 0% represent wild-type untreated activity at baseline. Means represented calculated from 7 independent repeats (Additional file 1: Figures S2, S3). *MT* mutant construct. *WT* wild-type construct, *tx* drug treated, *ut* untreated. **d** Total area under the curve (AUC) calculations for the MAP kinase pathway calculated using Graph Pad Prism. Also presented is the ratio of the AUC calculation of the tested EGFR L792 mutations and C797S-positive control versus T790M-negative control

To test the hypothesis that the L792F/H mutations confer resistance to osimertinib, we characterized the oncogenic activity of the mutants using a high-throughput functional in vitro assay [22]. Cells were transfected with *EGFR* expression constructs encoding an L858R sensitizing mutation, T790M resistance mutation, and one additional putative resistance mutation. Downstream signaling pathway activation—namely, MAPK/ERK and JAK-STAT—was assessed by measuring nuclear translocation of two reporters (ERK2 and STAT3) which shuttle from the cytoplasm to the nucleus upon pathway activation [22]. As expected, L858R/T790M induced activation of both MAPK/ERK and JAK-STAT pathways, which was inhibited by osimertinib in a dose-dependent manner (Fig. 2b, c). In contrast, L858R/T790M/C797S demonstrated resistance to osimertinib at all doses (p = 0.004 for the MAPK pathway and p = 0.004 for the JAK\STAT pathway, students t = test), compatible with irreversible loss of the osimertinib binding site. Importantly, the addition of L792H (p = 0.086 for the MAPK pathway, students t = test) and, to a lesser degree, L792F (p = 0.085 for the MAPK pathway, students t = test) to L858R/T790M induced intermediate levels of resistance that were overcome by increasing levels of osimertinib. This can be also seen in a 2-times higher AUC value as compared to L858R alone (Fig. 2d).

## Discussion

In this report, through clinical cfDNA NGS we identify *EGFR* L792 mutations in 22 of 1851 (1.2%) NSCLC patient cases with an *EGFR* T790M mutation. These L792 mutations appear to be a non-covalent mechanism of osimertinib resistance in which alterations in the EGFR ATP binding pocket diminish, but do not entirely prevent, osimertinib binding. In vitro assays suggest that increasing doses of osimertinib may overcome this resistance and inhibit EGFR activity, compatible with steric hindrance of drug binding or altered affinity to the drug or ATP rather than elimination of the binding site.

These results are consistent with recent reports of *EGFR* L792 mutations. Chen et al. [19] reported L792 mutations identified through cfDNA testing of plasma or pleural effusion in three patients with NSCLC progressing on osimertinib, with a follow-up study from the same group [20] identifying mutations at this residue in 11/93 (12%) of Chinese patients with osimertinib-resistant lung cancer.

There are inherent limitations to examining the prevalence of *EGFR* L792 mutations in the context of co-occurring T790M mutations. This approach was used due to the unavailability of treatment history details for genomic data from a commercial laboratory. With the recent approval of osimertinib for first-line use, this genomic context may not apply moving forward. Nishino et al. [23] found that *EGFR* L792 mutations in combination with L858R but in the absence of T790M conferred moderate resistance to osimertinib in vitro. Future studies examining the prevalence and functional effect of *EGFR* L792 mutations in the absence of T790M may clarify how broadly this data may be extrapolated in the dynamic landscape of drug approvals and treatment sequences.

Notably, the case described in detail above was found to have multiple *EGFR* mutations on cfDNA NGS, as did many other cases subsequently identified in the cohort prevalence analysis (Additional file 1: Table S2). The emergence of multiple alterations across the course of disease and treatment makes it increasingly difficult to delineate the isolated impact of any individual mutation in the acquired resistance process; this limitation of traditional analysis heightens the need for repeated comprehensive genomic profiling in the setting of clinical progression to capture the full context of changes under treatment pressure. The evolution of multiple on-target alterations underscores the complexity of the genomic landscape that can emerge in the setting of TKI resistance and highlights the importance of repeat genomic analysis, and in particular cfDNA NGS to non-invasively capture heterogeneous resistance, in detecting potentially targetable genomic alterations over the disease course.

## Supplementary information

**Additional file 1.** Supplementary figures and tables.

## Data Availability

The datasets generated and analyzed during the current study are not publicly available due to constraints given the origin of the genomic data from a clinical testing laboratory.
