# Supplementary material for: Identification of osimertinib-resistant EGFR L792 mutations by cfDNA sequencing: oncogenic activity assessment and prevalence in large cfDNA cohort
Source: Exp Hematol Oncol. 2019 Oct 11;8:24. doi: 10.1186/s40164-019-0148-7 (PMC6788107; doi:10.1186/s40164-019-0148-7)
Supplement: Supplementary file 1 — Additional file 1. Supplementary figures and tables. [file 40164_2019_148_MOESM1_ESM.docx]

**Additional file 1**

**Additional Methods**

*cfDNA sequencing*

Plasma cfDNA NGS studies were performed at a CLIA-, CAP-, NYSDOH-approved clinical laboratory (Guardant360, Guardant Health, Inc., Redwood City, CA). Whole blood was collected in Streck tubes and submitted to the laboratory, where cfDNA was extracted from plasma and analyzed by massively parallel paired-end synthesis by complete sequencing as previously described [1, 2]. This comprehensive genomic test allows for non-invasive detection of all major types of somatic alterations, including point mutations, indels, fusions, and copy number amplifications, of critical exons in 70-73 genes (Figure S2).

*Phylogenetic analysis*

*EGFR* variants identified by Guardant360 cfDNA NGS that occur within the length of a read (i.e., T790M, C797S, L792F, L792H, and F795C) were manually phased to identify unique molecules. The evolutionary relationship of unique molecules was inferred using a Dollo parsimony model [3].

*Structural modeling*

The locations of candidate resistance mutations were mapped to a publicly available EGFR crystal structure [4] using Jmol [5].

*In vitro functional characterization*

Functional experiments were performed in a CLIA-certified laboratory (NovellusDx, Jerusalem, Israel). *EGFR* mutant expression constructs were synthesized using the Q5 site-directed mutagenesis kit (New England Biolabs). Mutant *EGFR* expression constructs and EGFP-tagged ERK2 or STAT3 reporter constructs were transfected into HeLa cells using Fugene HD (Promega). Cells were incubated for 24 hours after transfection, fixed using paraformaldehyde 4%, counterstained with DAPI, and assessed for nuclear or cytoplasmic localization of the reporter construct using a Nikon Eclipse TI fluorescent microscope. Image segmentation algorithms were used to quantify these reporters in their active (nuclear) state [6]. For measurement of osimertinib effect, the drug at the indicated concentration (i.e., 2.5 nM, 10 nM, 40 nM, 156 nM, 625 nM, 2500 nM) was added for a period of 18 hours, starting 6 hours after transfection.

**Figure S1.** Guardant360 (A) 70-gene and (B) 73-gene panels. Gene names in bold indicate complete exonic sequencing, those not in bold indicate critical exonic sequencing.

A.

B.

**Figure S2.** Activity of the MAP Kinase pathway (ERK2–reporter). Independent repeats of averages shown in Fig. 2B of the functional activity of *EGFR* mutations compared to wild-type *EGFR*.


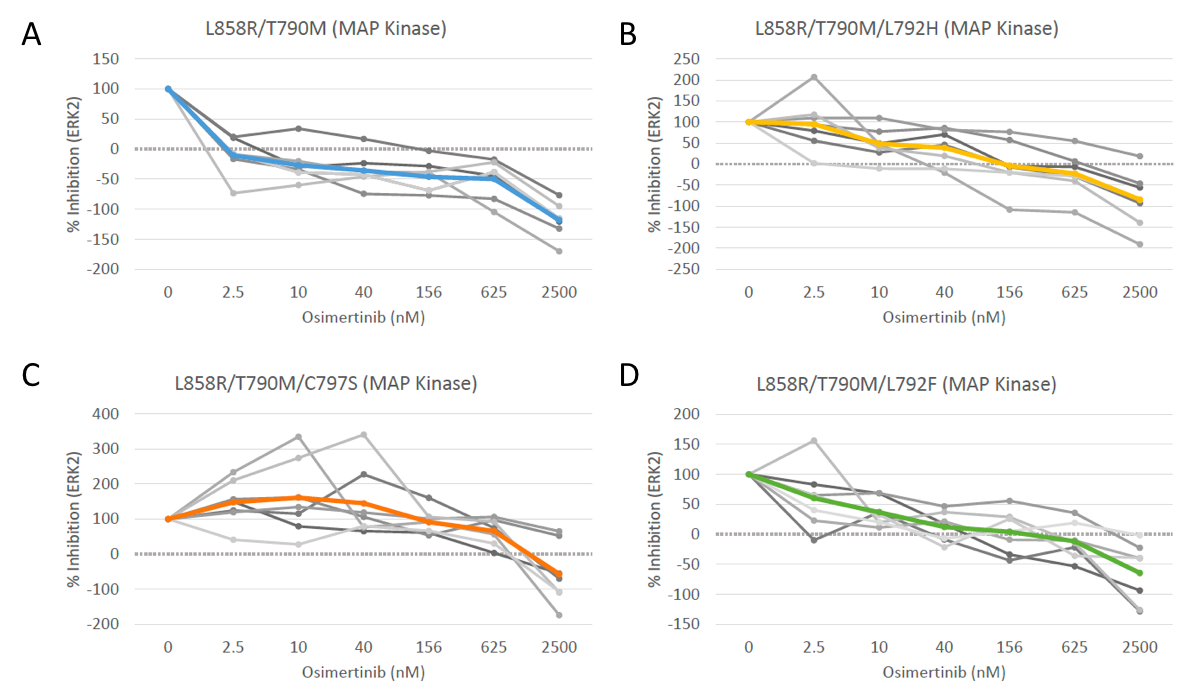


**Figure S3.** Activity of JAK-STAT pathway (STAT3–reporter). Independent repeats of averages shown in Fig. 2C of the functional activity of *EGFR* mutations compared to wild-type *EGFR*.


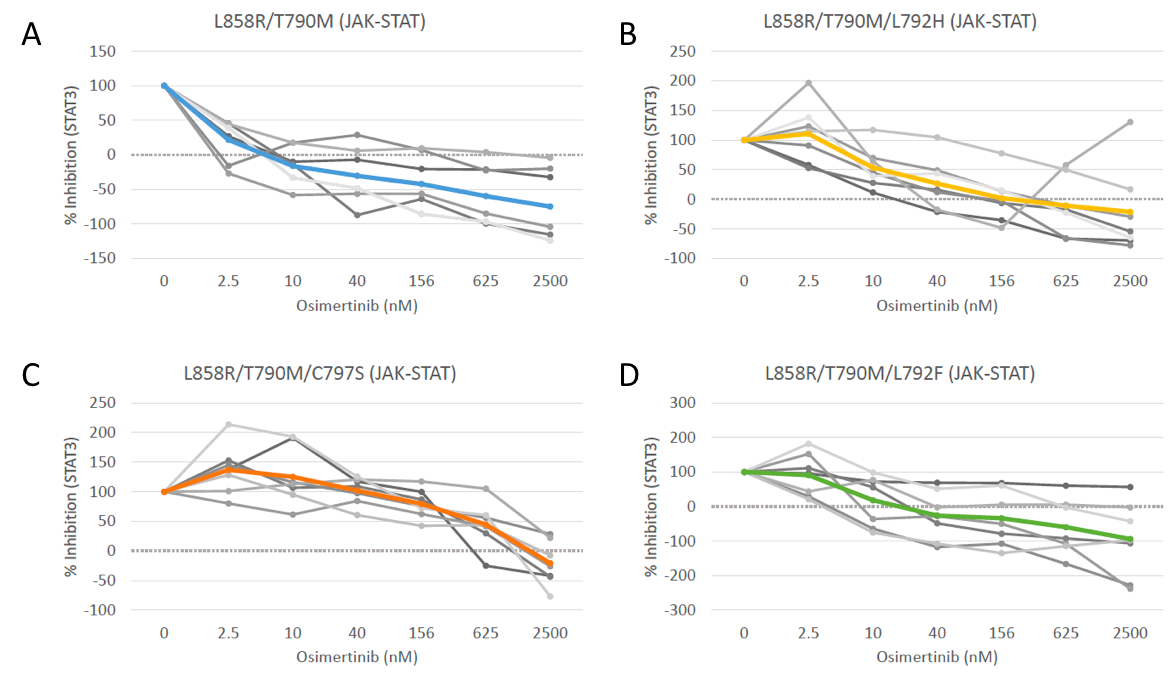


**Table S1.** Somatic alterations identified by Guardant360 cfDNA test in index patient described in detail, at the time of disease progression on osimertinib. AMP, amplification.

| Gene | Alteration | cDNA | cfDNA VAF (%) or Plasma Copy Number |
| --- | --- | --- | --- |
| *APC* | R232* | c.694C>T | 19.8% |
| *EGFR* | L858R | c.2573T>G | 16.92% |
| *EGFR* | T790M | c.2369C>T | 8.43% |
| *EGFR* | C797S | c.2389T>A | 4.57% |
| *EGFR* | L792H | c.2375T>A | 1.38% |
| *EGFR* | L718Q | c.2153T>A | 0.68% |
| *EGFR* | F795C | c.2384T>G | 0.35% |
| *BRCA1* | C1828Y | c.5483G>A | 0.14% |
| *EGFR* | L792F | c.2374C>T | 0.12% |
| *AR* | AMP | NA | 1.42 |
| *FGFR1* | AMP | NA | 2.48 |
| *CCND2* | AMP | NA | 2.50 |

**Table S2.** Multiple *EGFR* nonsynonymous alterations identified by cfDNA analysis in additional T790M- and L792-mutation positive samples. Del, deletion; AMP, amplification. *Initial patient whose case is described in detail.

| Patient | L792 variant(s) | Activating Mutation | C797S variant(s) | Other nonsynonymous *EGFR* alterations |
| --- | --- | --- | --- | --- |
| 1* | L792F (c.2374C>T), L792H (c.2375T>A) | L858R | c.2389T>A | L718Q, F795C |
| 2 | L792F (c.2374C>T) | L858R | none | none |
| 3 | L792H (c.2375T>A) | L858R | none | L718Q, G769S |
| 4 | L792H (c.2375T>A) | L858R | c.2389T>A, c.2390G>C | L718Q, G729V, G796S, V802F, G901G, R932G, P975L, AMP |
| 5 | L792H (c.2375T>A), L792V (c.2374C>G) | Exon 19 del | c.2390G>C | L718Q, I732_E736del, P741T, Q791E, P794T, F795L, G796S, G930del, Q951C, AMP |
| 6 | L792F (c.2374C>T) | Exon 19 del | c.2390G>C | none |
| 7 | L792F (c.2374C>T) | Exon 19 del | c.2390G>C, c.2389T>A | Q791H, AMP |
| 8 | L729H (c.2375T>A) | Exon 19 del | none | None |
| 9 | L792P (c.2375T>C) | L858R | none | G796A, AMP |
| 10 | L792F (c.2374C>T) | Exon 19 del | none | AMP |
| 11 | L792V (c.2374C>G), L792H (c.2375T>A) | L858R | none | L718V, AMP |
| 12 | L792H (c.2375T>A) | L858R | none | G630W, L718Q, L718V, AMP |
| 13 | L792H (c.2375T>A) | L858R | none | L718Q, AMP |
| 14 | L792H (c.2375T>A) | Exon 19 del | none | G724S, I744M, S811F, AMP |
| 15 | L792H (c.2375T>A) | Exon 19 del | c.2389T>A | L718Q, S768I, G796S, C797G, C797*, V802F, AMP |
| 16 | L792R (c.2375T>G) | L858R | none | L718Q, AMP |
| 17 | L792F (c.2374C>T), L792V (c.2374C>G) | Exon 19 del | c.2390G>C | G729V, AMP |
| 18 | L792H (c.2375T>A) | Exon 19 del | none | AMP |
| 19 | L792F (c.2374C>T) | Exon 19 del | c.2390G>C | G724S |
| 20 | L792P (c.2375T>C) | L858R | none | none |
| 21 | L792H (c.2375T>A) | Exon 19 del | c.2390G>C | none |
| 22 | L792F (c.2374C>T) | L858R | c.2389T>A, c.2390G>C | none |

**Table S3.** Phasing of *EGFR* L792 mutations relative to *EGFR* T790M and other *EGFR* osimertinib resistance mutations, when phasing was possible (C797S, G796S, and other L792 mutations).

|  | T790M | Osimertinib  Resistance Mutations |
| --- | --- | --- |
| *Cis* | 27 | 0 |
| *Trans* | 5 | 36 |

**References**

1. Lanman RB, Mortimer SA, Zill OA, Sebisanovic D, Lopez R, Blau S, et al. Analytical and Clinical Validation of a Digital Sequencing Panel for Quantitative, Highly Accurate Evaluation of Cell-Free Circulating Tumor DNA. PLoS ONE. 2015;10:e0140712

2. Odegaard JI, Vincent JJ, Mortimer S, Vowles JV, Ulrich BC, Banks KC, et al. Validation of a Plasma-Based Comprehensive Cancer Genotyping Assay Utilizing Orthogonal Tissue- and Plasma-Based Methodologies. Clin Cancer Res. 2018;24:3539–49.

3. Le Quesne WJ. The Uniquely Evolved Character Concept and its Cladistic Application. Systematic Zoology. 1974;23:513–7.

4. Yun C-H, Mengwasser KE, Toms AV, Woo MS, Greulich H, Wong K-K, et al. The T790M mutation in EGFR kinase causes drug resistance by increasing the affinity for ATP. Proc Natl Acad Sci U S A. 2008;105:2070–5.

5. Jmol: an open-source Java viewer for chemical structures in 3D.

6. Golbstein J, Tocker Y, Sharivkin R, Tarcic G, Vidne M. A Novel High-Throughput Multispectral Cell Segmentation Algorithm. In: Valdés Hernández M, González-Castro V, editors. Medical Image Understanding and Analysis. Springer International Publishing; 2017. p. 754–66.
